# Data for mitochondrial proteomic alterations in the aging mouse brain

**DOI:** 10.1016/j.dib.2015.05.004

**Published:** 2015-05-21

**Authors:** Kelly L. Stauch, Phillip R. Purnell, Lance M. Villeneuve, Howard S. Fox

**Affiliations:** Department of Pharmacology and Experimental Neuroscience, University of Nebraska Medical Center, Omaha, NE 68198, USA

**Keywords:** Mitochondria, Aging, Proteomics

## Abstract

Mitochondria are dynamic organelles critical for many cellular processes, including energy generation. Thus, mitochondrial dysfunction likely plays a role in the observed alterations in brain glucose metabolism during aging. Despite implications of mitochondrial alterations during brain aging, comprehensive quantitative proteomic studies remain limited. Therefore, to characterize the global age-associated mitochondrial proteomic changes in the brain, we analyzed mitochondria isolated from the brain of 5-, 12-, and 24-month old mice using quantitative mass spectrometry. We identified changes in the expression of proteins important for biological processes involved in the generation of precursor metabolites and energy through the breakdown of carbohydrates, lipids, and proteins. These results are significant because we identified age-associated proteomic changes suggestive of altered mitochondrial catabolic reactions during brain aging. The proteomic data described here can be found in the PRIDE Archive using the reference number PXD001370. A more comprehensive analysis of this data may be obtained from the article “Proteomic analysis and functional characterization of mouse brain mitochondria during aging reveal alterations in energy metabolism” in PROTEOMICS.

## Specifications table

**Table:** 

Subject area	Biology
More specific subject area	Aging
Type of data	Protein Expression Table
How data was acquired	Super-SILAC Mass Spectrometry; AB SCIEX Triple-TOF 5600; searched against the UniProtKB/Swiss-Prot database (Proteome ID UP000000589)
Data format	Normalized data
Experimental factors	Age
Experimental features	Brain mitochondria were isolated from male 5-, 12-, and 24-month mice (n=3) by differential centrifugation and immunomagnetic affinity isolation (crude mitochondrial preparations), the resulting protein lysate was used for mass spectrometry analysis using Data Dependent Acquisition (DDA) employing 50 dependent scans following each full scan
Data source location	University of Nebraska Medical Center, Omaha, NE
Data accessibility	The data are provided in the public PRIDE repository with the dataset identifier PXD001370. The direct URL to data is http://www.ebi.ac.uk/pride/archive/projects/PXD001370

## Value of the data

• These data describe the use of quantitative mass spectrometry-based proteomic experiments to assess the biological significance of mitochondrial alterations during brain aging.
• Age-associated alterations in the expression of mitochondrial proteins involved in key pathways important for energy metabolism were identified.
• Dynamic proteomic changes were identified between 5-, 12-, and 24-month of age in mouse brain mitochondria.

## 1 Data, experimental design, materials and methods

C57BL/6 mice from the National Institute on Aging were used for the proteomics experiments. Three male animals were used in the 5-, 12-, and 24-month groups. All experiments were conducted within NIH guidelines and approved by the University of Nebraska Medical Center IACUC.

### 1.1 Isolation of mouse brain mitochondria

Brains were isolated from 5-, 12-, and 24-month old mice using previously established methods [1]. Mitochondria were isolated from the brains as previously published using differential centrifugation and TOM22 immunomagnetic affinity isolation [1,2]. Mitochondrial protein amount was quantified using the Pierce 660 nm Protein Assay (Thermo Scientific). The isolated brain mitochondrial protein lysates (unlabeled) were combined in a 1:1 protein ratio with our previously described mitochondrial super-SILAC mix [3].

### 1.2 Sample preparation for mass spectrometry

Proteins (1:1 mixture of isolated brain mitochondrial lysates with the mitochondrial super-SILAC mix) were processed for mass spectrometry by trypsin digestion using the FASP method [4]. The resultant peptides were desalted using Oasis mixed-mode weak cation exchange cartridges (Waters) and dehydrated using a Savant ISS 110 SpeedVac Concentrator. Peptides were resuspended in 0.1% formic acid for LC-MS/MS analysis and quantified using a Nanodrop (Thermo Scientific) in conjunction with the Scopes method [5].

### 1.3 Mass-spectrometry based proteomics analysis

The peptide samples from each mouse (*n*=3, for each of the three ages (5-, 12-, and 24-month)) were analyzed in duplicate (two technical replicates) using a nano-LC-MS/MS in conjunction with an AB SCIEX TripleTOF 5600 instrument as previously described [1]. Peptide matching, protein identification, and relative protein quantitation (ratios of the amount of labeled (super-SILAC mix)-to-unlabeled (brain mitochondria samples) peptide) were performed using ProteinPilot (v.4.5). The search effort was set to “Thorough ID” and the False Discovery Rate (FDR) analysis was engaged, with the default setting for “Detected Protein Threshold (Unused ProtScore (Conf))” at >0.05 (10.0%). The criteria to exclude proteins from the analysis were as follows: FDR of 0.05 for both peptides and proteins, at least 6 amino acids must be contained by the protein, contaminants identified through the database search, and proteins identified in the reverse database as well as the additional cutoff values of Unused ProtScore≥1.3 and number of unique peptides ≥2. Quantitation was performed using the heavy super-SILAC mix as an internal standard and the resulting *H*/*L* ratios were normalized to this mix and expressed as light-to-heavy (*L*/*H*, sample/super-SILAC internal standards). The “ratio of ratio” value was determined, which is the change in protein expression from 5- to 12- or 12- to 24- or 5- to 24-month of age (proteomics data have been deposited to the ProteomeXchange consortium [6] (http://proteomecentral.proteomexchange.org) via the PRIDE partner repository with the dataset identifier PXD001370).

Overall, 1233 proteins were identified in all samples of mitochondria isolated from the comparative age groups of mice and quantification of these proteins indicated global proteomic changes during brain aging. The majority of the differentially expressed proteins were identified in energy generating catabolic pathways including glycolysis, mitochondrial β-oxidation, the pyruvate dehydrogenase complex, Kreb׳s cycle, the electron transport chain, and oxidative phosphorylation as well as protein degradation processes as highlighted in the interactive network diagrams created using Cytoscape version 3.1 [7]. Networks 1 and 2 display the proteins found to be differentially expressed from 5- to 12-month (216 proteins) and 12- to 24-month (190 proteins), respectively. The networks were created in Cytoscape using protein–protein interactions derived from the lists of differentially expressed proteins in the STRING: functional protein association networks database (string-db.org) [8]. These proteomics data revealed dynamic changes in brain mitochondrial protein expression during aging associated with an array of mitochondrial dependent catabolic pathways.

## Funding sources

This research was supported by the NIH Grants P30MH06221 and R01MH073490.

## Supporting information

Application 1

Application 2
